# Genomic characterization of high‐risk *Escherichia coli* and *Enterobacter hormaechei* clones recovered from a single tertiary‐care hospital in Pakistan

**DOI:** 10.1111/jam.15482

**Published:** 2022-02-21

**Authors:** Mashkoor Mohsin, Brekhna Hassan, Ali Usman Khan, Arslan Ali, Göte Swedberg, Badrul Hasan

**Affiliations:** ^1^ Institute of Microbiology University of Agriculture Faisalabad Pakistan; ^2^ Department of Medical Microbiology, School of Medicine Institute of Infection and Immunity, Cardiff University Cardiff UK; ^3^ District Headquarter Hospital Faisalabad Pakistan; ^4^ Section for Infectious Diseases, Department of Medical Sciences Uppsala University Uppsala Sweden; ^5^ Department of Medical Biochemistry and Microbiology, Uppsala Biomedical Center (BMC) Uppsala University Uppsala Sweden

**Keywords:** antimicrobial resistance, *bla*_NDM‐1_, Enterobacterales, ESBL, genomics, Pakistan

## Abstract

**Aims:**

Spread of carbapenem‐resistant Enterobacterales have become a global problem. We characterized extended‐spectrum β‐lactamase (ESBL)‐producing Enterobacterales from urinary tract infections cases from Allied Hospital Faisalabad, Pakistan.

**Methods and Results:**

Eleven (22%, 11/50) ESBL‐producing Enterobacterales (*Escherichia coli*; *n* = 10 and *Enterobacter hormaechei; n* = 1) were recovered and processed through VITEK‐2, PCR, rep‐PCR followed by whole‐genome sequencing (WGS) of ESBL‐producing *Ent. hormaechei* and carbapenem‐resistant *E. coli* isolates. Plasmid transferability of *bla*_NDM‐1_‐producers was assayed by conjugation experiments. All ESBL strains carried the *bla*_CTX‐M‐15_ gene. Of these *bla*_CTX‐M‐15_ producing *E. coli,* four also carried *bla*_NDM‐1_ located on transferable plasmids. All *E. coli* strains belonged to ST448 and displayed similar genetic features including genes for antimicrobial resistance, heavy metal, biocides and virulence. Genomic features of a multidrug‐resistant (MDR) *Ent. hormaechei* were also reported for the first time in Pakistan.

**Conclusion:**

Our findings indicate that *bla*_NDM‐1_ producing *E. coli* ST448 is a multidrug, heavy metals and biocides‐resistant strain. Therefore, the screening of these isolates may be effective in limiting the MDR bacteria spread in hospitalized patients and within the community.

**Significance and Impact of this Study:**

Spread of multi‐drug‐resistant ESBL‐producing bacteria in the clinical settings of Pakistan is a serious challenge and further limiting treatment options in the country. WGS could be used as a tool in the nationwide antibiotic surveillance programme to explore insights of spread and outbreak.

## INTRODUCTION

Antimicrobial resistance (AMR) is an ongoing public health problem of global dimensions. Over the last decades, multidrug‐resistant (MDR) Enterobacterales have become a serious public health issue as treatment options are minimizing day by day due to increased resistance to broad‐spectrum antibiotics such as cephalosporins and carbapenems (Bevan et al., 2017; Giske et al., 2009). Mainly, carbapenems have been serving as the last‐line drugs for MDR Gram‐negative bacteria treatment. An increased frequency of carbapenemase production in Enterobacterales family members has been recently reported in developing countries such as Bangladesh, Pakistan and India, where 1.9 billion people live under poor healthcare facilities (Choudhury et al., 2018; Hossain et al., 2020; Islam et al., 2012; Kumarasamy et al., 2010; Stewardson et al., 2019).

Extended‐spectrum β‐lactamases (ESBLs) is a rapidly emerging group of enzymes that can hydrolyze different generations of cephalosporins and carbapenems. Among different ESBLs, the production of ESBL_A_ and ESBL_CARBA_ enzymes are usually encoded by genes present on plasmids of members of the Enterobacterales family, and alongside they can carry several genes conferring resistance to other broad‐spectrum antibiotics, for example, aminoglycosides, macrolides and quinolones. The ESBLa group, known as classical ESBL, contains several *bla*_CTX‐M_‐β‐lactamase variants that can hydrolyze several generations of cephalosporins (e.g. third and fourth generations) (Bevan et al., 2017; Giske et al., 2009). The ESBL_CARBA_ group comprises different types of carbapenemase variants (*bla*_NDM_, *bla*_KPC_, *bla*_VIM_, *bla*_OXA‐48_ and *bla*_IMP_) that can hydrolyze antibiotics belonging to the carbapenem class (Giske et al., 2009). Historically, the Indian subcontinent has been considered as the hotspot for NDM (New Delhi‐metallo‐β‐lactamase [MBL])‐producing *Escherichia coli* (Giske et al., 2009; Kumarasamy et al., 2010). Alarmingly, carbapenem and/or cephalosporin resistance determinants like *bla*_CTX‐M_ and *bla*_NDM_ have been found in *E. coli* and *Enterobacter* species on top of other resistance mechanisms directed against fluoroquinolones and aminoglycosides (Al‐Agamy et al., 2019; Huang et al., 2019).

Biocide substances like silver and quaternary ammonium compounds (benzylkonium chloride, chlorhexidine and cetylpyridinium chloride) may also affect antibiotic resistance directly by selecting for porin deficiency and thereby it mediates a beta‐lactams cross‐resistance (Li et al., 1997; Shafaati et al., 2016). Alarmingly, *bla*_CTX‐M_‐producing *E. coli* started to show resistance to heavy metals and biocide compounds as these compounds are frequently used in hospitals, farms and agriculture to ensure medication, hygiene and biosecurity (Adamse et al., 2017; Shafaati et al., 2016; Silver, 2003; Sütterlin et al., 2014).

The increased prevalence of carbapenem‐resistant Enterobacterales has been reported sporadically from major hospitals in Pakistan in several studies and suggests spread in clinical settings in Pakistan where patients are coming from several locations of the country (Kumarasamy et al., 2010; Nahid et al., 2013; Qamar et al., 2019; Stewardson et al., 2019). However, there is also a lack of detailed genomic epidemiology of carbapenem‐resistant strains in Pakistani hospitals. Currently, little is known about the prevalence and genetic characteristics of clinical ESBL producing *E. coli* and *Enterobacter* strains co‐harbouring carbapenem resistance in Pakistan. In this study, we investigated the detailed molecular and genomic epidemiology of carbapenem‐resistant Enterobacterales isolated from hospitalized patients in a tertiary care hospital of Pakistan.

## MATERIALS AND METHODS

### Sample collection and isolation of ESBL producers

From January to March 2016, 50 clinical samples (urine, *n* = 40 and wound, *n* = 10) were collected in an ongoing study for the screening of ESBL producing Enterobacterales from Allied Hospital Faisalabad, Pakistan. Samples were streaked on CHROMagar‐ESBL (CHROMagarCo.) and incubated overnight at 37°C. One potential ESBL‐producer was selected from each plate for further investigations. Species identification of the ESBL‐producers was confirmed by API 20E biochemical test (bioMérieux) followed by MALDI‐TOF/MS (Bruker Daltonics) according to the manufacturer’s instruction.

The Institutional Bioethics/Biosafety Committee of the University of Agriculture, Faisalabad, has approved this study (D. No. 109/ORIC).

### Antimicrobial susceptibility testing

Confirmation of the ESBL production was done by double‐disc synergy test according to the CLSI guidelines (CLSI, 2016) followed by Vitek‐2 compact system (AST‐card GN38; bioMérieux). The Vitek‐2 system was also used for the detection of additional phenotypic AMR. MDR was defined as resistance to three or more different classes of antimicrobials. Carbapenemase production and their types were determined phenotypically by MBL&KPC&OXA‐48 discs kit (Liofilchem) according to the manufacturer’s instruction.

### Detection of ESBL and MBL producing genes by PCR

A Maxwell® 16 Cell DNA Purification Kit (Promega) was used with the automated Maxwell 16 SEV instrument in order to get purified genomic DNA from freshly harvested bacteria. Genomic DNA extracted from both methods was centrifuged for 10 min at 13,000 rpm and the supernatant was taken to be storaged at −20°C. A PCR protocol was used to detect ESBL genes *bla*_TEM_ *, bla*_*S*HV_ and *bla*_CTX‐M_ (Tofteland et al., 2007). DNA sequencing was performed on *bla*_CTX‐M_ positive samples to determine the *bla*_CTX‐M_ genotype. The screening of MBL genes was also performed by various real time‐PCRs for *bla*_VIM_, *bla*_NDM_, *bla*_IMP_, *bla*_GIM_, *bla*_SPM_ and *bla*_SIM_ (Swayne et al., 2013). ESBL‐producing isolates were screened for *bla*_OXA‐48_ using a method described previously(Poirel et al., 2011).

### Bacterial conjugation experiments

ESBL_CARBA‐_producing *E. coli* strains were tested to check their plasmid transferability by conjugation. As donors, four *bla*_NDM‐*1*_‐producing *E. coli* strains were analysed for conjugation. *E. coli* DA11782 (*mcrA, Δ(mrr‐hsdRMS‐mcrBC)*, *ΔlacX74*, *deoR*, *recA1*, *araD139Δ(ara‐leu)7697*, *galK*, *rpsL*, *endA1*, *nupG*, *rif* ^*R*^) was used as the recipient. Equal amounts of donor and recipient from overnight cultures in Luria‐Bertani broth were mixed and incubated without shaking overnight at 37°C. Approximately 10^9^ colony forming unit of conjugation mixture was plated on selective culture media plates containing 8 μg/ml meropenem and 100 μg/ml rifampin, and incubated overnight at 37°C.

### Whole‐genome sequencing and bioinformatics analysis

*Enterobacter hormaechei* and all *bla*_NDM_ positive *E. coli* strains were subjected to whole‐genome sequencing (WGS). Extraction of genomic DNA was performed with EZ1 Advanced XL system (QIAGEN) and fresh overnight stains cultured on blood agar plate were used for extraction. The extracted genomic DNA was measured using Qubit dsDNA assay kit (Life Technologies). Extracted genomic DNA was sequenced on Illumina platform (Illumina HiSeq 2500, 2 × 100 paired‐end) at the Science for Life laboratory (SciLifelab), generating 2 × 100 paired‐end sequences. De novo assembly was done using CLC Genomics Workbench version 12.0.3. WGS analysis was performed using open access bioinformatics web tools at Center for Genomic Epidemiology (CGE; www.genomicepidemiology.org) for detection of antibiotic resistance and biocide resistance genes, virulence genes and plasmid replicon types. Multilocus sequence typing was performed web‐tools from www.enterobase.warwick.ac.uk *E. coli* Multilocus sequence typing (MLST) Database. Detection of metal (Mercury; *mer* gene) and biocide (Silver; *sil*) resistance genes were performed using BLASTn search from the assembled whole‐genome sequence data using web‐based open source platform named Galaxy (https://usegalaxy.org/). Geneious Prime (v. February 3, 2019) was used to get information about the structure of *bla*_NDM‐1_ gene and the reference sequence used to annotate the *bla*_NDM‐1_ contigs was KP770033. Single nucleotide polymorphisms (SNPs) in the isolates core genome were identified by Parsnp (Harvest suite v.1.0) (Treangen et al., 2014) using internal reference (EC8). All the *bla*_NDM‐1_ positive *E. coli* isolates were analysed and compared with 11 global ST448 *E. coli* genomic sequences of human clinical origin taken from Enterobase database (www.enterobase.warwick.ac.uk). The phylogenetic analyses of these genomic sequences were performed with CSI‐Phylogeny v 1.4 (https://cge.cbs.dtu.dk/services/CSIPhylogeny). The acquired antibiotic resistance genes of these isolates were analysed using ResFinder. The phylogenetic tree was visualized using interactive tree of life (iTOL v 6) (Letunic & Bork, 2016).

## RESULTS

**FIGURE 1:**
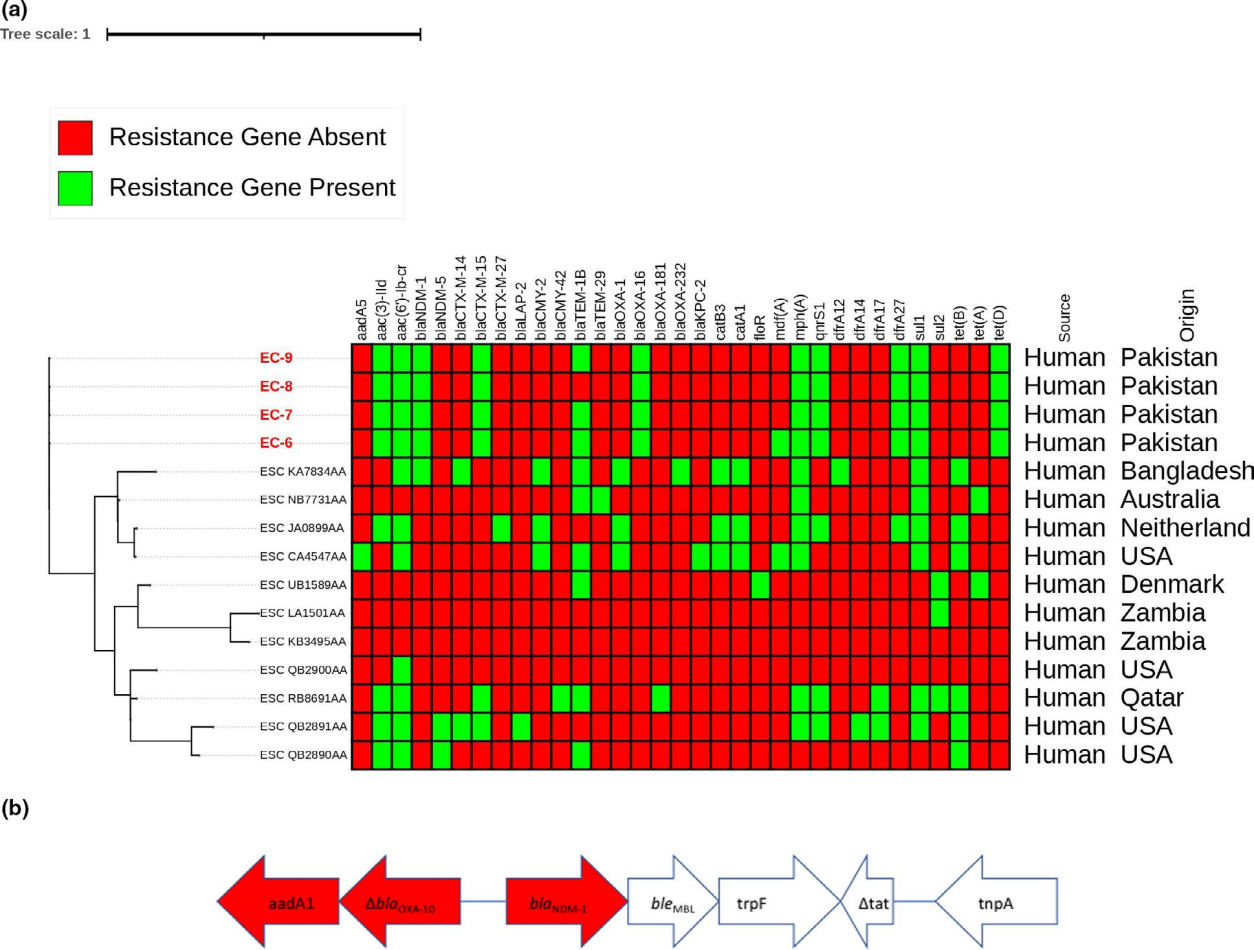
(a) Phylogenomic comparison of *Esherichia coli* strains to belong to ST448. The maximum likelihood tree was created using CSI‐phylogeny v 1.4 and visualized in interactive tree of life (iTol v6). (b) Genetic context surrounding *bla*‐_NDM‐1_ gene. Genes are denoted by arrows. Antimicrobial resistance genes are coloured red, mobile elements genes and other features are colourless

In total, 11 strains were found as ESBL positive phenotypically consisting of *E. coli* (*n* = 10) and *Ent. hormaechei* (*n* = 1). ESBL‐producers were found as MDR and displayed phenotypic resistances from 4 to 6 different classes of antibiotics. All strains were resistant to ampicillin, enrofloxacin and marbofloxacin (Table 1). Only four *E. coli* strains were resistant to carbapenems for example, meropenem and imipenem. Phenotypic diversity of antibiotic resistance is presented in Table 1. All ESBL producing isolates were positive for *bla*_CTX‐M‐I_ and genes encoding for the *bla*_CTX‐M‐II_, *bla*_CTX‐M‐III_ and *bla*_CTX‐M‐IV_ groups were not detected. Sanger sequencing of *bla*_CTX‐M‐I_ positives PCR amplicons revealed the *bla*_CTX‐M‐15_ genotype. Four *E. coli* strains were positive for the *bla*_NDM_ gene, but none of the samples were positive for *bla*_VIM_, *bla*_IMP_, *bla*_GIM_, *bla*_SPM_, *bla*_SIM_ and *bla*_OXA‐48_. Sanger sequencing of *bla*_NDM_ positives PCR amplicons revealed the *bla*_NDM‐1_ genotype. Strong co‐relation exists between antibiotic resistance phenotypes and genotypes among the resistant strains. One of the ESBL‐producing *E. coli* isolates was found positive by the O25b‐ST131 PCR assay. The rep‐PCR analysis of all *E. coli* strains identified six different clonal types (A, B, C, D, E, F). All *bla*_NDM‐1_‐producing *E. coli* strains belonged to type A and appeared as the dominant clonal type (Table 1). WGS analysis of *bla*_NDM_ positive strains revealed *bla*_NDM‐1_ in addition to *bla*_CTX‐M‐15_ type. All *bla*_NDM‐1_ positive *E. coli* strains were carrying similar antibiotic resistance markers (Figure 1). Resistance markers in *E. coli* strains included β‐lactams(*bla*_OXA‐16_, *bla*_TEM‐1B_), carbapenems (*bla*_NDM‐1_), quinolones (*qnrS1*, *aac[6′]‐Ib‐cr*), aminoglycosides (*aadA1, aad16, aac[3]‐IId*), tetracycline (*tetD*), macrolide (*mphA*), sulfonamides (*sul1, sul2*) and trimethoprim (*dfrA27*). Analysis of genetic environment confirmed the *bla*_NDM‐1_ gene was adjacent to the *ble*_MBL_ gene (a belomycin resistance protein) followed by a truncated *trpF* gene downstream, on the other hand, *aadA1* conferring aminoglycoside resistance and a truncated narrow‐spectrum β‐lactamase, *bla*_OXA‐10_ was located in the upstream direction (Figure 1).

**TABLE 1:** Phenotypic and genotypic characteristics of *Escherichia coli* and *Enterobacter hormaechei* recovered from a single tertiary‐care hospital in Pakistan

Sample ID	Sample origin	Species	Additional resistances	Rep‐PCR profile	ESBL and MBL genotypes
EC‐5	Urine	*E. coli*	AM, GM, TM, DC, TE, ENR, MRB, SXT	B	*bla*_CTX‐M‐15_
EC‐6	Urine	*E. coli*	AM, GM, TM, DC, TE, ENR, MRB, SXT, IPM, MP	A	*bla*_CTX‐M‐15_, *bla*_NDM‐1_
EC‐7	Urine	*E. coli*	AM, GM, TM, DC, TE, ENR, MRB, SXT, IPM, MP	A	*bla*_CTX‐M‐15_, *bla*_NDM‐1_
EC‐8	Urine	*E. coli*	AM, GM, TM, DC, TE, ENR, MRB, SXT, IPM, MP	A	*bla*_CTX‐M‐15_, *bla*_NDM‐1_
EC‐9	Urine	*E. coli*	AM, GM, TM, DC, TE, ENR, MRB, SXT, IPM, MP	A	*bla*_CTX‐M‐15_, *bla*_NDM‐1_
EC‐11	Urine	*E. coli*	AM, GM, TM, ENR, MRB, SXT	B	*bla*_CTX‐M‐15_
EC‐12	Urine	*E. coli*	AM, C, DC, TE, ENR, MRB, SXT	C	*bla*_CTX‐M‐15_
EC‐25	Wound	*E. coli*	AM, GM, TM, DC, TE, ENR, MRB, SXT, C	F	*bla*_CTX‐M‐15_
EC‐14	Urine	*E. coli*	AM, TM, DC, TE, ENR, MRB	D	*bla*_CTX‐M‐15_
EC‐15	Urine	*E. coli*	AM, GM, TM, DC, TE, ENR, MRB	E	*bla*_CTX‐M‐15_
EC‐13	Urine	*Ent. hormaechei*	AM, GM, TM, ENR, MRB, SXT	‐^a^	*bla*_CTX‐M‐15_, *bla*_ACT‐24_

Abbreviations: AMP, ampicillin; C, chloramphenicol; CO, colistin, DC, doxycycline; ENR, enrofloxacin; ESBL, extended‐spectrum β‐lactamase; GM, gentamicin; IPM, imipenem; MBL, metallo‐β‐lactamase; MP, meropenem; MRB, marbofloxacin; SXT, PO, polymyxin, sulfamethoxazole/trimethoprim; TE, tetracycline; TM, tobramycin.

^a^ Not done.

No silver resistance gene was found; however, several mercury resistance genes (*merC* and *merR*) were found. All *bla*_NDM‐1_‐producing *E. coli* strains carried *qacE* genes responsible for resistance to different quaternary ammonium compounds such as benzylkonium chloride, chlorhexidine, and cetylpyridinium chloride (Table S1).

All *bla*_NDM‐1_‐producing *E. coli* strains belonged to phylogroupB1. Characterization of *fimH* is an important typing scheme in *E. coli* as this is an adhesin‐related gene that has virulence potential. All sequenced *bla*_NDM‐*1*_‐producing *E. coli* carried *fimH35*. In total, three different clinically associated important virulence genes were found in the *bla*_NDM‐1_ positive *E. coli* strains; *gad* (glutamate decarboxylase), *terC* (tellurium iron resistance) and *lpfA* (long polar fimbriae) (Table S1). The wgSNP analysis of *bla*_NDM‐1_‐producing *E. coli* strains showed that EC‐6, EC‐7, EC‐8 and EC‐9 were highly closely related with SNP differences of ≤9. Phylogenetic comparison of *E. coli* ST448 with global collection of human origin ST448 showed that these strains are phylogenetically distinct (Figure 1).

MLST analysis of the *bla*_NDM‐1_‐producing strains identified them as ST448 complex (Acthman scheme), however, core‐genome MLST revealed three different STs (74,416, 76,469 and 100,030) indicating genome‐wide diversity. All *bla*_NDM‐1_‐producing *E. coli* strains had transferable plasmids as confirmed by the conjugation experiment. Plasmid replicon types IncX3, IncFII(Yp), and ColpVC were found in all *bla*_NDM‐1_‐producing *E. coli* isolates.

*Enterobacter hormaechei* carried resistant markers for cephalosporins (*bla*_CTX‐M‐15_), β‐lactams (*bla*_OXA‐1_, *bla*_ACT‐24)_, quinolones *(qnrB6*, *qnrB32*, *aac[6′]‐Ib‐cr*), aminoglycosides (*aac[3]‐IIa*), phenicol (*catB3*), sulfonamides (*sul1*), trimethoprim (*dfrA27*) and fosfomycin (*fosA2)*. On the other hand, *Ent. hormaechei* had 2 different plasmid replicon types: IncX3 and IncFIB (pHCM2).

## DISCUSSION

During winter months complicated urinary tract and wound infections produced by the members of the Enterobacterales is considered a serious challenge in hospital settings in Pakistan because of the amount of failed treatment. Increased prevalence of ESBL‐producing Enterobacterales has been reported in Pakistani hospitals involving mostly *E. coli* (Abrar et al., 2018). Alarmingly some of the ESBL‐producing *E. coli* isolates were resistant to carbapenems, aminoglycosides and quinolones. The ESBL situation was getting worse because of the MDR features to other non‐β‐lactams antibiotics displayed by these pathogens, ultimately limiting the treatment options available in Pakistani hospitals. Both, hospital and non‐hospital uses of antibiotics in Pakistan are not regulated but indiscriminated; some essential antibiotics like cephalosporins, carbapenems, macrolides, quinolones and aminoglycosides are extensively used regularly in clinical practice (Saleem et al., 2019). As a consequence, *E. coli* isolates from our study were resistant to these essential antibiotics indicating how overuse and misuse of essential antibiotics potentially impacted on clinical isolates.

Genetic characterization of ESBL‐producing *E. coli* isolates confirmed the presence of *bla*_CTX‐M‐15_ genotype in all isolates. *E. coli* isolates phenotypically resistant to meropemen and imipenem carried the *bla*_NDM‐1_ genotype in addition to *bla*_CTX‐M‐15_. Epidemiology typing by rep‐PCR confirmed all *E. coli* harbouring *bla*_NDM‐1_ to belong to one clonal profile (A). The rest of the *E. coli* isolates were completely different from each other. Several reports previously confirmed the abundance of *bla*_CTX‐M‐15_ in clinical and environmental isolates in Pakistan (Rafaque et al., 2018; Umair et al., 2019). Also, *bla*_NDM‐1_ has started to be endemic among ESBL‐producing Gram negatives including *E. coli* (Qamar et al., 2019). Interestingly, the *bla*_NDM‐1_ positive *E. coli* isolates were carrying other extended‐spectrum β‐lactams resistance genes, for example, *bla*_OXA‐10_ which we reported for the first time in *E. coli* from Pakistan. The *bla*_OXA‐10_ gene was reported in *Pseudomonas aeruginosa* from Pakistan and Turkey (Danel et al., 1998; Ullah et al., 2017). *E. coli* isolates carrying *bla*_NDM‐1_ also carried several‐resistant markers for quinolones, aminoglycosides, macrolides, biocides and antifolates, typical for multidrug resistance plasmids. Generally, *bla*_NDM‐1_ producing bacteria display resistance to the members of antibiotics classes carbapenems, aminoglycosides and quinolones (Kumarasamy et al., 2010). All *bla*_NDM‐1_ positive *E. coli* belonged to B1 confirming the increased clinical importance of this phylogroup in Pakistan. Thus, a certain *bla*_NDM‐1_‐producing *E. coli* clone is infecting and circulating among the patients in the tertiary‐care centre of Pakistan. Several studies from Pakistan reported the dominance of ESBL‐producing *E. coli* B2 phylogroup generally associated with urinary tract infection (Bashir et al., 2012; Shafaati et al., 2016).

MLST analysis of *bla*_NDM‐1_‐producing *E. coli* confirmed them as belonging to the ST448 complex. *E. coli* ST448 known as uropathogenic strain is believed to have evolved from common intestinal strains and reported in several countries in Asia and Africa including neighbouring India and Bangladesh (Abd El Ghany et al., 2018; Choudhury et al., 2018; Hossain et al., 2020; Muggeo et al., 2020). PCR‐based replicon typing detected 3 different plasmid replicon‐types; IncX3, IncFII(Yp) and ColpVC. IncFII is a common plasmid type among members of the Enterobacterales family mostly in *E. coli* which is able to carry *bla*_NDM‐1_ genotype (Pedersen et al., 2018; Rozwandowicz et al., 2018). Highly conjugative IncX3 plasmid has been very successful in *bla*_NDM‐_genotype spread in *E. coli* from several countries in Asia, Africa and Europe. This is the first study exploring the association between plasmid and *bla*_NDM‐1_ genotype in clinical *E. coli* strains from Pakistan. Also, IncX3 plasmid started to appear in the ESBL‐producing clinical isolates. All the *bla*_NDM‐1_ *E. coli* were closely related with SNP differences of less than 9. This could be the result of a recent transmission event considering the estimated mutation rate (1 SNP/genome/year) and SNP cut‐off for outbreaks (<10 SNPs) among *E. coli* (Schürch et al., 2018).

Virulence factors can often be associated with plasmids and resistant bacteria can become more virulent because of MDR plasmids (Ramirez et al., 2014). In this study, *bla*_NDM‐1_‐producing *E. coli* were carrying several virulence factors that are associated with clinical outcomes. The most striking examples are the following: (1) *terC*, which was previously reported in ESBL‐producing *E. coli* infected patients, (2) *gad*, a significant pathogenic marker described in different types of human‐associated *E. coli* infections which can survive in stomach acidic environment to colonize hosts, (3) *ipfA*, assisting in bacterial adhesion associated with intestinal colonization and progression of infection and (4) *fimH35* which is an adhesin‐related gene that has virulence potential (Al‐Farsi et al., 2020; Grant et al., 2001; Zhou et al., 2019). Thus, the *bla*_NDM‐1_‐producing *E. coli* recovered from urine samples are uropathogenic virulent *E. coli* which could potentially impact the clinical outcomes. Interestingly, *bla*_NDM‐1_‐producing *E. coli* were carrying resistance markers for mercury (e.g, *merC* and *merR*) and Quaternary ammonium compounds (e.g. *qacE*). A study from Iran reported the presence of *qacE* gene in *E. coli* from urine samples and these genes could be responsible for an efflux pump that can contribute to antibiotic resistance (Shafaati et al., 2016). Thus, the presence of biocide resistance indicates an alarming situation with no easy solution in sight. This is the first study reporting the potential metal and biocide resistance in *bla*_NDM‐1_‐producing bacterial strains in Pakistan.

*Enterobacter hormaechei* is an emerging pathogen, and a key member of the highly diverse *Ent. cloaceae* complex has been reported to carry ESBL genes (Gou et al., 2020). In this study, one isolate causing urinary tract infection was *Ent. hormaechei* carrying a wide range of resistant markers, that is *bla*_CTX‐M‐15_, *bla*_OXA‐1_, *bla*_ACT‐24_, *qnrB6*, *qnrB32*, *aac(6′)‐Ib‐cr*, *aac(3)‐IIa*, *catB3*, *sul1*, *dfrA27* and *fosA2*. This is the first study from Pakistan reporting the presence of MDR *Ent. hormaechei* isolated from the clinical samples. The ESBL‐producing *Ent. hormaechei* carried two different plasmid replicon types (IncX3 and IncFIB) that are strongly associated with carbapenemase‐producing genes like *bla*_NDM_ and *bla*_KPC_.

One of the main limitations of our study is that it was conducted in only single tertiary care hospital in Faisalabad using a small sample size which may not represent the whole population. Seasonal trend may also affect the result. These limitations could be resolved in future studies of CRE in Pakistan.

## CONCLUSIONS

Based on the findings of the present study, it can be concluded that ESBL_Carba_ is gradually increasing in Pakistan with co‐resistance to other classes of essential antibiotic classes. The *E. coli* strains carrying *bla*_NDM‐1_ and *Ent. hormaechei* carrying *bla*_ACT‐24_ are high‐risk clones that are present in the hospitals of Pakistan and these strains are resistant to biocides. Antibiotic resistance is a serious challenge in Pakistani hospitals and there is no easy solution in sight.

## CONFLICT OF INTEREST

The authors declare no competing financial interest exists.

## Supporting information


Table S1
