# Supplementary material for: Genomic characterization of high‐risk Escherichia coli and Enterobacter hormaechei clones recovered from a single tertiary‐care hospital in Pakistan
Source: J Appl Microbiol. 2022 Feb 21;132(5):3907–14. doi: 10.1111/jam.15482 (PMC9306472; doi:10.1111/jam.15482)
Supplement: Supplementary file 1 — Table S1 [file JAM-132-3907-s001.docx]

| **Sample**  **ID** | **Sample**  **Origin** | **Species** | **Virulence genes** | **Metal resistance genes** |
| --- | --- | --- | --- | --- |
| EC-6 | Urine | *E. coli* | *gad*, *terC, lpfA* | *qacE, merC, merR* |
| EC-7 | Urine | *E. coli* | *gad*, *terC, lpfA* | *qacE, merC, merR* |
| EC-8 | Urine | *E. coli* | *gad*, *terC, lpfA* | *qacE, merC, merR* |
| EC-9 | Urine | *E. coli* | *gad*, *terC, lpfA* | *qacE, merC, merR* |

Table S1: Virulence and metal resistance genes profile of bla_NDM-1_ positive *E. coli* strains from this study

*gad,* (glutamate decarboxylase); *terC,* (tellurium iron resistance) *lpfA,* (long polar fimbriae), *merC* and *merR*, (mercury resistance); *qacE*, quaternary ammonium compounds
